# Quantifying and reducing statistical uncertainty in sample-based health program costing studies in low- and middle-income countries

**DOI:** 10.1177/2050312118765602

**Published:** 2018-03-22

**Authors:** Claudia L Rivera-Rodriguez, Stephen Resch, Sebastien Haneuse

**Affiliations:** 1 Department of Statistics, The University of Auckland, Auckland, New Zealand; 2 Department of Health Policy and Management, Harvard T.H. Chan School of Public Health, Boston, MA, USA; 3 Department of Biostatistics, Harvard T.H. Chan School of Public Health, Boston, MA, USA

**Keywords:** Costing studies, immunization, study design, treatment programs

## Abstract

**Objectives:**

In many low- and middle-income countries, the costs of delivering public health programs such as for HIV/AIDS, nutrition, and immunization are not routinely tracked. A number of recent studies have sought to estimate program costs on the basis of detailed information collected on a subsample of facilities. While unbiased estimates can be obtained via accurate measurement and appropriate analyses, they are subject to statistical uncertainty. Quantification of this uncertainty, for example, via standard errors and/or 95% confidence intervals, provides important contextual information for decision-makers and for the design of future costing studies. While other forms of uncertainty, such as that due to model misspecification, are considered and can be investigated through sensitivity analyses, statistical uncertainty is often not reported in studies estimating the total program costs. This may be due to a lack of awareness/understanding of (1) the technical details regarding uncertainty estimation and (2) the availability of software with which to calculate uncertainty for estimators resulting from complex surveys. We provide an overview of statistical uncertainty in the context of complex costing surveys, emphasizing the various potential specific sources that contribute to overall uncertainty.

**Methods:**

We describe how analysts can compute measures of uncertainty, either via appropriately derived formulae or through resampling techniques such as the bootstrap. We also provide an overview of calibration as a means of using additional auxiliary information that is readily available for the entire program, such as the total number of doses administered, to decrease uncertainty and thereby improve decision-making and the planning of future studies.

**Results:**

A recent study of the national program for routine immunization in Honduras shows that uncertainty can be reduced by using information available prior to the study. This method can not only be used when estimating the total cost of delivering established health programs but also to decrease uncertainty when the interest lies in assessing the incremental effect of an intervention.

**Conclusion:**

Measures of statistical uncertainty associated with survey-based estimates of program costs, such as standard errors and 95% confidence intervals, provide important contextual information for health policy decision-making and key inputs for the design of future costing studies. Such measures are often not reported, possibly because of technical challenges associated with their calculation and a lack of awareness of appropriate software. Modern statistical analysis methods for survey data, such as calibration, provide a means to exploit additional information that is readily available but was not used in the design of the study to significantly improve the estimation of total cost through the reduction of statistical uncertainty.

## Introduction

The global health community has become increasingly concerned with resource needs and financial sustainability as health programs in low- and middle-income countries (LMICs) expand coverage to include new treatment choices into routine practice, build capacity for long-term growth of their health systems, and plan for transition away from international donor support.^1–5^ This, in turn, has led to an increase in demand for rigorous program costing studies.^6–8^ Recently, a number of high-profile projects sought to estimate service delivery costs by sampling and surveying health facilities.^9–12^ With this backdrop, the Expanded Program on Immunization Costing and Financing^13^ project, supported by the Bill and Melinda Gates Foundation and in partnership with local organizations, sought to obtain detailed costing information for routine child immunization^14^ from six LMICs that expanded their immunization programs with financial support from Gavi, the Vaccine Alliance.^13,15^ Using standardized definitions, data collection procedures, and analytic methods, these studies estimated the total program costs for the year 2011, as well as the cost per dose delivered and the cost per fully immunized child. Although specific details varied across the six studies, the sampling was performed using a combination of purposeful and random selection at the various levels. In Honduras, for example, the final study sample consisted of 71 of 1534 facilities, from 8 of 20 health regions.^16^ Average costs at each level, calculated on the basis of information collected within the sampling frame, were then weighted to obtain estimates of the total national costs for routine immunization (RI).

While the methods adopted by the European Prospective Investigation into Cancer and Nutrition (EPIC) have many strengths, the original analyses and reports published estimates of total costs although it generally did not report measures that quantify statistical uncertainty due to the fact that only a subsample of facilities were included in the survey, such as standard errors or 95% confidence intervals (CIs).^16^ Although the reporting of these types of measures is standard in the public health and medical literature, they are often not reported in health service costing studies based on data from a subsample of service delivery points; the EPIC studies are, therefore, not unusual in this regard. That uncertainty is not reported may be due, in part, to the difficulties associated with managing complex sample designs and generating appropriate sample weights, as well as a lack of awareness/understanding of techniques that can be used to compute them. Distinct from other sources of uncertainty, such as that due to model^12^ specification for which sensitivity analyses are available,^17^ understanding and quantifying statistical uncertainty is critical in that it provides important contextual information for decision-makers, which may have implications for planning and budgeting, and for prioritizing future costing studies.

Against this backdrop, this article provides an overview of statistical uncertainty in the context of complex survey-based costing studies as well as methods for quantification. In addition, we describe recently developed statistical methods that exploit auxiliary information to reduce uncertainty, thereby substantially improving the precision of the final estimates. Throughout, we illustrate the ideas using data from the Honduran EPIC study.

## RI in Honduras

In 2012–2013, the Honduran Ministry of Health, in partnership with the Pan American Health Organization, undertook a study to estimate the total economic costs of RI provided in 2011.^16^ At the time RI was delivered through a network of 1534 public and non-governmental organizations (NGOs) health facilities across the country, organized geographically into 298 municipalities within 20 health regions (Table 1). Administratively, the program was overseen locally through designated EPI “lead” facilities (one per municipality), regional offices (one per region), and centrally through offices in the capital, Tegucigalpa.

**Table 1.** Number of municipalities and health facilities within the 20 health regions in Honduras in 2011. Also shown are the number of municipalities and health facilities that were selected by the Honduran EPIC study.

	Municipalities		Health facilities	
	Total *N_M: r_*	Selected *n_M: r_*	Total *N_F: r, m_*	Selected *n_Fm,: r_*
Health region				
Atlántida	8	3	54	9
Choluteca	16	3	148	9
Colón	10	0	65	0
Comayagua	21	0	92	0
Copán	23	0	79	0
Cortés	11	3	63	9
El Paraíso	19	0	101	0
Francisco Morazán	27	3	96	8
Gracias a Dios	6	0	49	0
Intibucá	17	0	56	0
Islas de la Bahía	4	0	8	0
La Paz	19	0	68	0
Lempira	28	3	95	9
Ocotepeque	16	0	45	0
Olancho	23	3	171	9
Santa Bárbara	28	0	85	0
San Pedro Sula	1	1	19	9
Tegucigalpa	1	1	69	9
Valle	9	0	77	0
Yoro	11	0	94	0
Total	298	20	1534	71

EPIC: European Prospective Investigation into Cancer and Nutrition.

Given the multilevel structure of the program in Honduras, costs associated with RI accrued centrally, at the level of the health region and at the level of the health facility. To estimate the total cost of the RI program (i.e. across all levels), a survey was conducted to collect detailed resource use and cost information from the sample of 71 health facilities within eight of the health regions (Table 1). Relevant data were also collected from the regional offices for the eight selected regions, as well as from the central administrative offices. Two of the selected regions were San Pedro Sula and Tegucigalpa, both populous metropolitan regions that are geographically small so that the health facilities (19 and 69, respectively) were clustered into a single municipality in each. The remaining six regions were purposefully chosen to reflect the level of urbanization, socioeconomic status, and geography of the country as a whole.

From each of the eight selected regions, between 8 and 9 health facilities were selected. In San Pedro Sula and Tegucigalpa, the EPI lead facility (responsible for coordinating the immunization program in the municipality) was chosen along with eight additional facilities selected via stratified random sampling based on facility type. For the six other regions, three municipalities were selected in each on the basis of the size of the population below 1 year old. Within each municipality, the EPI lead facility was purposefully chosen along with 1–2 non-lead facilities selected via stratified random sampling on the basis of facility type.

## Estimation based on data from a sample survey

As indicated in the Introduction, costing studies may have many goals. To help simplify the exposition, we present the methods in the context of estimating total costs of RI in Honduras. The ideas are generally applicable to any costing study goal, however, and to emphasize this, we also present results pertaining to the average facility-level cost per dose. Throughout, all results were obtained using the survey package for the freely available R statistical computing language.^18^ The online Supplementary Materialsdocument provides the actual code used to generate the results, along with additional technical details.

### Expressing total cost

To make the task at hand more concrete, we write the total cost of RI in Honduras as $T=T_{C}+T_{R}+T_{F}$, where $T_{F}$ represents the total RI costs accrued at all 1534 health facilities, $T_{R}$ represents the (additional) total RI costs accrued at the 20 regional offices, and $T_{C}$ represents the (additional) total RI costs accrued centrally. In expressing the total cost this way, the task of estimation can be broken down into three (arguably simpler) subtasks. To highlight the fact that $T_{R}$ represents the totality of costs accrued at the regional level, we write it as

$$T_{R}=\overset{N_{R}}{\underset{r=1}{\sum }}T_{R:r}$$

where $T_{R:r}$ is the total RI regional-office accrued costs specific to region *r* and $N_{R}$ is the number of regions. Note that $N_{R}$ = 20 in Honduras. Similarly, one can represent $T_{F}$ as

$$T_{F}=\overset{N_{R}}{\underset{r=1}{\sum }}\overset{N_{M:r}}{\underset{m=1}{\sum }}\overset{N_{F:rm}}{\underset{f=1}{\sum }}T_{F:rmf}$$

where $T_{F:rmf}$ is the total RI facility-accrued costs specific to facility *f* in municipality *m* in region *r*. Note that the notation has been developed to acknowledge that the number of municipalities within any given region, denoted $N_{M:r}$ for region *r*, may vary across regions and that the number of facilities within any given municipality, $N_{F:rm}$ for municipality *m* in region *r*, may also vary (see Table 1). Finally, since $T_{C}$ represents costs accrued from a single source (i.e. the central administrative offices), there is no need to adopt a decomposition that is analogous to those adopted for $T_{F}$ and $T_{R}$.

### Estimation via inverse-probability weighting

In the absence of complete data from all facilities/regions, neither $T_{F}$ nor $T_{R}$ can be directly calculated. As such, these components of the total cost T must be estimated. Standard approaches will fail to produce a valid estimate for the entire population of interest. The reason is that complex sampling designs use different selection probabilities for different units. One approach to obtaining a correct estimate is to use *inverse-probability weighting* (IPW).^18,19^ Focusing on $T_{R}$, although there are many ways of expressing the IPW estimate, one useful approach is to write

$$\hat{TR}=\overset{N_{R}}{\underset{r=1}{\sum }}I_{r}\times W_{r}\times T_{R:r}$$

where $I_{r}$ is an indicator of whether the region *r* was selected and $W_{r}=1/\pi_{r}$, with $\pi_{r}$ as the probability that region *r* was selected to be part of the survey. Note that in the Honduran EPIC study, 12 of the 20 regions have $I_{r}=0$ so that they will not directly contribute to $\hat{TR}$. However, the remaining eight regions for which $I_{r}=1$ will directly contribute, with their contributions (i.e. the observed value of $T_{R:r}$) up-weighted by the inverse of *πr* $\pi_{r}$ to account for the fact that there is missing data from the 12 regions that were not selected. Intuitively, by up-weighting the contributions from those regions that were selected by the IPW estimate, $\hat{TR}$ is attempting to recover the total that would have been calculated had complete data from all regions been readily available. An analogous expression for the IPW estimate of $T_{F}$, denoted as $\hat{TF}$, is given in the online Supplementary Materials.

### Results

Given estimates $\hat{TR}$ and $\hat{TF}$, the IPW estimate of the total cost of RI in Honduras is $\hat{T}=T_{C}+\hat{TR}+\hat{TF}$. From the second column in Table 2, the IPW estimate of the total costs for RI in Honduras is 2011 of $\hat{T}=\mathrm{US}\$35.9\;\mathrm{millions}$. The weights used in this calculation were those used by. In addition, Table 2 provides IPW estimates of the component costs. For example, the estimated total labor costs associated were US$20.3 million, while the estimated total cold chain costs were US$1.5 million.

**Table 2.** Estimated costs of RI in Honduras in 2011 based on standard and calibrated IPW (also shown are estimated SE and 95% CIs).^16^

	Standard IPW (US$ in millions)					Calibrated IPW^a^ (US$ in millions)				
	Estimate	Formula based		Bootstrap based^b^		Estimate	Formula based		Bootstrap based^b^	
		SE	95% CI	SE	95% CI		SE	95% CI	SE	95% CI
Total	35.9	2.7	(30.7, 41.2)	2.7	(31.2, 41.4)	31.9	1.6	(28.9, 35.0)	2.0	(29.68, 37.46)
Component source										
Vaccine and supplies	8.0	–	–	–	–	8.0	–	–	–	–
Labor	20.3	2.2	(16.1, 24.6)	2.2	(16.4, 24.8)	17.8	1.1	(15.7, 20.0)	1.3	(16.0, 21.1)
Volunteers	0.9	0.2	(0.4, 1.3)	0.2	(0.5, 1.3)	0.7	0.2	(0.4, 1.1)	0.2	(0.4, 1.2)
Cold chain	1.5	0.3	(1.0, 2.0)	0.3	(1.0, 2.1)	1.2	0.1	(1.0, 1.4)	0.1	(1.0, 1.5)
Vehicles	0.3	0.1	(0.1, 0.6)	0.1	(0.2, 0.6)	0.3	0.1	(0.2, 0.4)	0.1	(0.2, 0.4)
Buildings	1.1	0.1	(0.9, 1.4)	0.1	(0.9, 1.4)	1.0	0.1	(0.8, 1.1)	0.1	(0.8, 1.1)
Other	1.6	0.0	(1.6, 1.7)	0.0	(1.6, 1.7)	1.5	0.1	(1.3, 1.7)	0.2	(1.4, 2.0)
Per diem	2.1	0.2	(1.7, 2.6)	0.3	(1.7, 2.6)	1.8	0.1	(1.6, 2.1)	0.2	(1.6, 2.2)

IPW: inverse-probability weighting; SE: standard errors; CI: confidence interval.

b Calibration based on the total number of doses administered and the number of facilities by type/size.

c Quantile-based 95% bootstrap confidence interval.

From the second column of Table 3, we see that the IPW estimate of the (overall) average facility-level cost per dose is US$5.52. Also shown are the corresponding estimates by facility size and type. Consistent with the results reported by Janusz et al.,^16^ the average cost per dose is substantially higher among smaller facilities than among larger facilities, with estimates ranging from US$2.99 per dose in huge facilities to US$44.62 in tiny facilities.

**Table 3.** Estimated average facility-level cost per dose by facility size and type based on standard and calibrated IPW (also shown are estimated SE and 95% CI).^16^

	Standard IPW (US$)					Calibrated IPW^a^ (US$)				
	Estimate	Formula based		Bootstrap based^b^		Estimate	Formula based		Bootstrap based^b^	
		SE	95% CI	SE	95% CI		SE	95% CI	SE	95% CI
Overall	5.52	0.63	(4.29, 6.75)	0.64	(4.39, 6.82)	4.76	0.27	(4.23, 5.29)	0.30	(4.22, 5.54)
Facility size^c^										
Huge	2.99	0.99	(1.05, 4.93)	0.99	(1.31, 5.2)	2.42	0.16	(2.11, 2.73)	0.30	(2.03, 3.02)
Large	1.33	0.74	(–0.12, 2.78)	0.69	(0.27, 2.93)	3.39	1.12	(1.19, 5.59)	1.60	(2.07, 8.52)
Medium	4.81	1.27	(2.32, 7.30)	1.28	(2.45, 7.42)	5.24	0.72	(3.83, 6.65)	0.70	(4.05, 6.93)
Small	12.21	2.53	(7.25, 17.17)	2.54	(7.6, 17.39)	8.68	0.91	(6.90, 10.46)	0.90	(7.02, 10.5)
Tiny	44.62	29.98	(0, 103.38)	30.40	(2.75, 112.15)	24.56	5.26	(14.25, 34.87)	6.10	(14.45, 34.54)
Facility type										
CESAMO	5.44	0.99	(3.50, 7.38)	0.96	(3.64, 7.31)	5.20	0.45	(4.32, 6.08)	0.60	(4.32, 6.58)
CESAR	8.45	2.01	(4.51, 12.39)	2.03	(5.08, 12.81)	6.41	0.54	(5.35, 7.47)	0.60	(5.27, 7.64)
Hospital	1.49	0.89	(0, 3.23)	0.89	(0, 3.23)	1.21	0.59	(0.05, 2.37)	0.80	(0.00, 2.98)

IPW: inverse-probability weighting; SE: standard errors; CI: confidence interval.

CESAMO: Centro de Salud con Médico y Odontólogo—these are health centers typically found in more densely populated areas.

CESAR: Centro de Salud Rural—these are health centers usually found in rural areas.

a Calibration based on the total number of doses and the number of facilities per type and size.

b Quantile-based 95% bootstrap confidence interval.

c Sizes in number of doses: huge ≥10,000; large 5000–9999; medium 1500–4999; small 500–1499; tiny <500.

## Uncertainty in costing studies

While the second columns of Tables 2 and 3 fulfill the primary goals of providing estimates of total RI costs and average facility-level costs per dose, the fact that the values are estimates that are subject to statistical uncertainty should not be ignored. Given the structure of the Honduran RI program and the design of the EPIC survey, three sources of statistical uncertainty must be considered: (1) uncertainty due to only 8 of 20 regions having been selected, (2) uncertainty due to only 20 of 298 municipalities having been selected, and (3) uncertainty due to only 71 of 1534 facilities having been selected. Note that if the design of the Honduran EPIC survey had been such that a complete enumeration at any of these levels had been incorporated, then that particular source would not contribute to the overall uncertainty. This would be the case, for example, if the survey had selected at least one facility from each of the 20 regions (instead of just 8).

### Quantification and estimation of uncertainty

In statistical analyses, uncertainty is typically quantified using the standard error (the square-root of the variance of the estimate), which is then used to calculate a 95% CI.^20^ For a given population structure and study design for the survey, an analytic expression (i.e. formula) for the variance of the IPW estimate can be derived and implemented. In the online Supplementary Materials document, for example, we provide a detailed derivation specific to the variance of $\hat{T}$ in the Honduran context. In many instances, however, deriving formulae can be challenging and researchers can instead take advantage of a number of software options that have general implementations appropriate for a wide range of settings, including the survey package in R, the “svy” commands in STATA, and the SURVEY procedure in SAS.

When the number of surveyed sampling units (i.e. facilities) is small, as is the case in many costing studies, the theory that underpins the validity of analytic formulae may not hold with a resulting mischaracterization of uncertainty. As an alternative, analysts may use a resampling-based technique such as the bootstrap.^14,21,22^ The online Supplementary Materials document provides an algorithm for bootstrap-based estimation of the standard error and a 95% CI for $\hat{T}$. We also note that the bootstrap is available as an option for most analyses in the survey package for R.^18^

### Results

Columns 3–6 of Table 2 present estimated standard errors and corresponding 95% CIs for the IPW-based estimates of total costs for RI in Honduras based on both the analytic expressions and the bootstrap. We see, for example, that the estimated standard error for the total cost based on either approach is US$2.7 million. Furthermore, the formula-based 95% CI (US$30.7 million and US$41.2 million) provides a concrete characterization of uncertainty associated with the IPW estimate of total cost.

Standard error estimates and corresponding 95% CIs for the component costs are also provided. For example, the formula-based 95% CI for the total labor cost is US$16.1 million and US$24.6 million. Note that the standard error for the total vaccine costs is US$0 because these are solely accrued by the central administrative offices, are therefore known, and do not need to be estimated.

Similarly, columns 3–6 of Table 3 present estimates of statistical uncertainty for the average facility-specific cost per dose. We see, for example, that the formula-based 95% CI for the overall average facility-specific cost per dose is US$4.29 and US$6.75, while the corresponding 95% CI for CESAR facilities is US$4.51 and US$12.39. Interestingly, some of the formula-based intervals include negative values of average facility-specific cost per dose. For example, the 95% CI for facilities with fewer than 500 doses per year is −US$14.14 and US$103.38. Clearly, negative values are not possible indicating a possible breakdown of the theory for the estimation of average facility-specific cost per dose for this type of facility; indeed, only 3 of the 71 facilities administered fewer than 500 doses per year. From column 6, however, we see that the corresponding bootstrap-based 95% CI is US$2.75 and US$112.72, which does not include any negative values and, therefore, provides more reasonable/interpretable results.

## Leveraging auxiliary information to reduce uncertainty

While IPW estimators provide a means of accounting for the sampling scheme in a survey, they are well known to be statistically inefficient in the sense that they tend to exhibit greater uncertainty than other estimators of the same quantity. One approach to improving statistical efficiency, thereby reducing uncertainty, and making 95% CIs tighter is to use auxiliary information that is readily available on all regions/facilities. In the context of a costing study for RI, one possibility would be to use information on the total number of doses administered in each facility; these totals have been shown to be highly correlated with the total costs. Suppose that this information is readily available, one could use it as a basis for stratifying the facilities *a priori*, that is, before they are selected by the design.

If the auxiliary information is available, but was not used to inform the sampling scheme, analysts have at their disposal a range of methods that permit its use post hoc. One such method is calibration, the essential idea of which is to use the auxiliary information to modify the weights used in the standard IPW estimator in such a way that uncertainty is reduced.^23^ To see this, let $D_{F}$ denote the known total number of doses administered across all 1534 facilities. Although $D_{F}$ is known, one could nevertheless write down the IPW estimator based solely on the survey data from 71 facilities

$$\hat{D_{F}}=\overset{N_{R}}{\underset{r=1}{\sum }}\overset{N_{M:r}}{\underset{m=1}{\sum }}\overset{N_{F:rm}}{\underset{f=1}{\sum }}I_{rmf}\times W_{rmf}\times D_{F:rmf}$$

Ostensibly, the $W_{rmf}$ weights can and should be the same as those used as in $\hat{T_{F}}$ above, since they reflect the design that gave rise to the observed data. Given that $D_{F}$ is actually known, however, one can calculate the discrepancy (or distance) between $D_{F}$ and its estimate $\hat{D_{F}}$. Furthermore, one can work to reduce this distance by modifying the weights to force $\hat{D_{F}}$ to equal $D_{F}$. In doing so, the resulting IPW estimate $\hat{D_{F}}$ has zero variance (since it is being forced to equal the known total). The modified weights, also referred to as *calibrated weights*, can then be used to form a new IPW estimator of T. In theory, the variance of this new estimator will never be larger than the variance of the IPW estimator based on the original weights. The extent to which the variance is reduced, however, depends on the strength of the association between the auxiliary information and the quantity being estimated. An important caveat is that the calibrated weights may no longer be representative of the original design so that the calibrated IPW estimator may exhibit a small amount of bias. As a general post hoc analysis technique, calibration seeks to balance the reductions in variance obtained by folding in known totals into the estimation with the potential bias that arises when the weights are modified.

### Methods

We used information from Honduras on the number of doses delivered annually at each health facility to perform post hoc calibration. The R code for this, based on the survey package for R, is given in the Supplementary Materials.

### Results

The right-hand parts of Tables 2 and 3 present the results. We see from Table 2, for example, that while there is a modest change in the estimated total cost, from US$35.9 to US$31.9 million, there is a substantial reduction in uncertainty, with the formula-based calibrated IPW standard error estimated to be US$1.6 million, down from US$2.7 million. This, in turn, results in a 95% CI that is 42% tighter, indicating the improvement in accuracy. While the corresponding bootstrap estimate is somewhat higher (US$2.0 million), it is still smaller than its counterpart for the standard IPW estimate.

In addition, the point estimate for large facilities is smaller than for huge facilities, whereas for the rest of categories the cost follows an increasing trend when the facility size decreases. This inconsistency, however, is addressed and corrected by the calibrated methods as observed in Table 3.

## Discussion

When analyzing data from survey-based costing studies in LMICs, researchers must contend with a range of types of uncertainty. One such type is the uncertainty associated with the model, the essential question being “do the results/conclusions change as one changes the model that is basis for the analysis?” Toward investigating this type of uncertainty, researchers may employ sensitivity analyses.^17,24^ Another type of uncertainty is statistical uncertainty, the essential question being “how different would the results/conclusions have been if a different sub-sample had been selected?” We focus on the latter in this article. Understanding and quantifying this type of uncertainty provides critical information for decision-making. For example, stratifying by facility size could have yield more efficient estimators as the cost varies significantly across the sizes. As such, studies estimating the incremental cost of health technologies or interventions report CIs for regression models that use patient-level data. Likewise, reports of analyses of complex population health surveys such as the Demographic and Health Surveys (IMF Macro) routinely include CIs. Studies aimed at measuring the full delivery cost of health services in LMICs, however, have to-date not systematically reported measures of statistical uncertainty alongside point estimates. Moreover, when they are included, the methods used usually do not leverage auxiliary information that is often available to reduce uncertainty. The calibration method not only provides a means of gaining efficiency in the estimation of the total delivery costs of health programs, but also in studies where the primary interest is to evaluate the incremental cost of a novel intervention.^1,5^ We believe that with a greater awareness of these tools and opportunities, together with the references provided, analysts and policy-makers will have more concrete information on which to base decisions as well as to design future costing studies.

## Conclusion

Measures of uncertainty associated with survey-based estimates of program costs, such as standard errors and 95% CIs, provide important contextual information for health policy decision-making and key inputs for the design of future costing studies. Such measures are often not reported, possibly because of technical challenges associated with their calculation and a lack of awareness of appropriate software. Modern statistical analysis methods for survey data, such as calibration, provide a means to exploit additional information that is readily available but was not used in the design of the study to significantly improve the estimation of total cost through the reduction of uncertainty.

## Supplemental Material

SAGE_SM – Supplemental material for Quantifying and reducing statistical uncertainty in sample-based health program costing studies in low- and middle-income countries Click here for additional data file. Supplemental material, SAGE_SM for Quantifying and reducing statistical uncertainty in sample-based health program costing studies in low- and middle-income countries by Claudia L Rivera-Rodriguez, Stephen Resch and Sebastien Haneuse in SAGE Open Medicine
