# Supplementary material for: Quantifying and reducing statistical uncertainty in sample-based health program costing studies in low- and middle-income countries
Source: SAGE Open Med. 2018 Mar 22;6:2050312118765602. doi: 10.1177/2050312118765602 (PMC5888835; doi:10.1177/2050312118765602)
Supplement: SAGE_SM – Supplemental material for Quantifying and reducing statistical uncertainty in sample-based health program costing studies in low- and middle-income countries [file SAGE_SM.docx]

Supplementary Materials for:

Quantifying and reducing uncertainty in sample-based health program costing studies in low-and-middle income countries

This document provides additional technical details, specifically:

A complete summary of notation needed for estimation and inference in the context of the Epic Honduran study.

Expression for the IPW estimate of $T_{F}$.

Detailed representations of the IPW estimator of total cost and its’ corresponding variance.

An overview of the bootstrap as a means to estimate standard errors and construct 95% confidence intervals.

**Section A**

Table SM-1. Summary of notation critical for design purposes

| Level | Quantity | Interpretation |
| --- | --- | --- |
|  |  |  |
| Region | $N_{R}$ | Total number of regions  * equal to 20 in Honduras |
|  | $n_{R}$ | Total number of regions selected by the design  * equal to 8 in Honduras |
|  | $S_{R}^{2}$ | Variance of the $N_{R}$ region-specific total costs |
|  |  |  |
| Municipality | $N_{M:r}$ | Total number of municipalities in region *r*  *** see column 1 of Table 1 |
|  | $n_{M:r}$ | Total number of municipalities in region *r* selected by the design  * see column 2 of Table 1 |
|  | $S_{M:r}^{2}$ | Variance of the $N_{M:r}$ municipality-specific costs within region *r* |
|  |  |  |
| Facility | $N_{F:r,m}$ | Total number of health facilities in municipality *m* in region *r*  * see column 3 of Table 1 |
|  | $n_{F:r,m}$ | Total number of health facilities in municipality *m* in region *r* selected by the design  * see column 4 of Table 1 |
|  | $S_{F:r,m}^{2}$ | Variance of the $N_{F:r,m}$ health facility-specific costs within municipality *m* in region *r* |

**Section B**

Analogous to the expression given in the main manuscript for the IPW estimate of $T_{R}$, one can express the IPW estimate of $T_{F}$ as:

$$\hat{T_{F}}= \sum_{r=1}^{N_{R}} \sum_{m=1}^{N_{M:r}} \sum_{f=1}^{N_{F:rm}} I_{rmf} \times{W_{rmf} \times T}_{F:rmf}$$

where $I_{rmf}$ is an indicator of whether facility *f* in municipality *m* in region *r* was selected, and ${W_{rmf}=1/\pi}_{rmf}$ with $\pi_{rmf}$ the probability that the same facility was selected. Similar to $\hat{T_{R}}$, only the 71 facilities with $I_{rmf}$=1 would directly contribute to $\hat{T_{F}}$, with their contributions up-weighted to account for the fact that detailed costing information is missing for the remaining facilities.

**Section C**

Expression for total cost

The total cost is $T=T_{C}+T_{R}+ T_{F}$ where

$$T_{F}= \sum_{r=1}^{N_{R}} \sum_{m=1}^{N_{M:r}} \sum_{f=1}^{N_{F:r,m}} T_{F:rmf}$$

with $T_{F:rmf}$ denoting the total RI cost specific to facility *f* in municipality *m* in region *r*, and

$$T_{R}= \sum_{r=1}^{N_{R}} T_{R:r}$$

with $T_{R:r}$ denoting is the total RI cost accrued at the regional office for the *r*^th^ region. Combining these components, the total cost is given by:

$$T=T_{C} + \sum_{r=1}^{N_{R}} T_{R:r}+ \sum_{r=1}^{N_{R}} \sum_{m=1}^{N_{M:r}} \sum_{f=1}^{N_{F:r,m}} T_{F:rmf}$$

which can be re-written as:

$$T=T_{C} + \sum_{r=1}^{N_{R}} \sum_{m=1}^{N_{M:r}} \sum_{f=1}^{N_{F:r,m}} U_{rmf}$$

where

$$U_{rmf}=T_{F:rmf}+ \left( T_{R:r} \times\frac{W_{r}}{W_{rmf}}\times\frac{1}{n_{M:r}}\times\frac{1}{n_{F:r,m}} \right)$$

is the total RI cost accrued by facility *f* in municipality *m* in region *r*, folding in the costs accrued at the regional level proportionally.

Expression for the variance of the IPW estimate of total cost

If we denote the IPW estimate of $T$ by $\hat{T}$, the variance can be calculated as:

$$V\left[ \hat{T} \right]= V_{R}+V_{M}+ V_{F}$$

where $V_{R}$ is the uncertainty in $\hat{T}$ due to only eight of 20 regions having been selected, $V_{M}$ is the uncertainty in $\hat{T}$ due to only 20 of 298 municipalities having been selected, and $V_{F}$ is the uncertainty in $\hat{T}$ due to only 71 of 298 facilities having been selected. Note, although the decompositions for $T$ (i.e. $T=T_{C}+T_{R}+T_{F}$) and $V\left[ \hat{T} \right]$ each have three components, the components do not align. Indeed each component of $V\left[ \hat{T} \right]$ represents the totality of uncertainty across all three components of $T$ due to sub-sampling at some specific level in the design.

Assuming random sampling of the regions, $V_{R}$ is:

$$V_{R}= \left( \frac{1}{n_{R}}-\frac{1}{N_{R}} \right) N_{R}^{2}S_{R}^{2}$$

where $S_{R}^{2}$ is the variance of the totals across the $N_{R}$ regions. Note, if all $N_{R}$ had been selected (i.e. $n_{R}$=$N_{R}$) then $V_{R}$ is equal to zero indicating that there is no uncertainty at the level of the region. Furthermore, if $S_{R}^{2}$=0 then $V_{R}$ is also equal to zero; this situation arises when the total region-specific costs do not vary. If $n_{R}$<$N_{R}$ and $S_{R}^{2}$>0, we see that the contribution to the uncertainty depends on the interplay between $n_{R}$, $N_{R}$, and $S_{R}^{2}$.

Similarly, assuming random sampling of municipalities within regions, $V_{M}$ is:

$$V_{M}= \frac{N_{R}}{n_{R}}\sum_{r\in P_{R}} \left( \frac{1}{n_{M:r}}-\frac{1}{N_{M:r}} \right) N_{M:r}^{2}S_{M:r}^{2}$$

where $P_{R}$ is the set of regions that is selected by the sampling scheme, and $S_{M:r}^{2}$ is the variance of the totals across the $N_{M:r}$ municipalities in region *r*. Note, there are $N_{R}$ values of $S_{M:r}^{2}$ (i.e. one per region).

Finally, assuming random sampling of facilities within municipalities, $V_{F}$ is:

$$V_{F}= \frac{N_{R}}{n_{R}}\sum_{r\in P_{R}} \frac{N_{M:r}}{n_{M:r}}\left\{ \sum_{m\in P_{M:r}} \left( \frac{1}{n_{F:r,m}}-\frac{1}{N_{F:r,m}} \right) N_{F:r,m}^{2}S_{F:r,m}^{2} \right\}$$

where $P_{M:r}$ is the set of municipalities within region *r* that is selected by the sampling scheme, and $S_{F:r,m}^{2}$ is the variance of the facility-specific costs across the $N_{F:r,m}$ facilities in municipality *m* in region *r*. Note, there are $N_{M:r}$ values of $S_{F:r,m}^{2}$ in region *r* (i.e. one per municipality).

**Section D**

The bootstrap method for the variance of the estimate of total cost

In practice use of analytic formulae as a means to compute the variance of an estimate, such as those given above, may be problematic. In particular, in some settings it may be difficult to derive an analytic expression for the variance that accurately reflects the sampling design or not even possible if the design is not well-defined. The latter can happen when aspects of the sampling scheme are not fully documented or if non-response is high so that the final sample cannot be said to arise from the design as planned. In other settings use of analytic formulae may yield confidence interval estimates that include negative values of total cost. While total cost clearly cannot be negative, this phenomenon may nevertheless occur either because standard error is very large and/or the central limit theorem may not hold for the given sample size.

In such settings it will still be desirable to report a confidence interval that is valid (in the sense that it attains the nominal coverage probability) but that is also interpretable (in the sense of corresponding to meaningful values). One option for doing so is to use the bootstrap, a resampling-based technique that does not require analytic formulae for the variance such as those given above ^1^. Here, following Rao and colleagues ^2^, we provide an algorithm for obtaining bootstrap-based estimates of a 95% confidence interval for estimates of total cost based on a sampling design that is stratified by regions/municipalities.

1. Within each stratum defined by region and municipality select a random sample with replacement of $n_{F:r,m}-1$ facilities. Refer to the totality of the selected facilities as the *b*^th^ bootstrap sample.
2. For each facility in this sample, calculate the bootstrap weight:

$$\tilde{w}_{rmf}^{b}= \left( \frac{n_{F:r,m}}{n_{F:r,m}-1} \right){m_{rmf}^{b}w}_{rmf},$$

where $m_{rmf}^{b}$ is the number of times that facility $rmf$ is selected in the bootstrap sample. Note, $\tilde{w}_{rmf}^{b}$ is defined to be zero for those facilities that are not included in the bootstrap sample. Furthermore, if the bootstrap is being performed for a calibrated estimator of the total cost then the $\tilde{w}_{rmf}^{b}$ weights in the bootstrap sample should be calibrated at this point.

1. Use the collection of new weights $\tilde{w}_{rmf}^{b}$ and the sample *b* to calculate the bootstrap estimator for the total cost:

$$\hat{T_{b}}= \sum_{r\in P_{R}} \sum_{m\in P_{M:r}} {\tilde{w}_{rmf}^{b}u}_{rm}^{b}$$

2. Repeat steps 1- 3 *B* times. While rules of thumb often suggest that values of *B*=100-500 will suffice Monte Carlo theory suggest that the larger the *B*, the more precise the results*.*
3. The bootstrap estimate is then:

$\hat{T}_{B}= \frac{1}{B}\sum_{b=1}^{B} \hat{T}_{b}$,

with a consistent estimator of its variance given by:

$\hat{V}\left( \hat{T}_{B} \right)= \frac{1}{B-1}\sum_{b=1}^{B} \left( \hat{T}_{b}-\hat{T}_{B} \right)^{2}$.

1. Two options are available to obtain confidence intervals:
2. Calculate a standard confidence interval $\hat{T_{b}}\pm z_{1-\alpha}\sqrt{\hat{V}\left( \hat{T}_{B} \right)}$.
3. Calculate a confidence interval based on the empirical distribution across the $B$ bootstrap samples. In this case, a confidence interval based on the quantiles can be used: $\left( q\left( \hat{T}_{b},\frac{\alpha}{2} \right), q\left( \hat{T}_{b}, 1-\frac{\alpha}{2} \right) \right)$

Finally, we note that the bootstrap is theoretically only appropriate when the first stage of sampling is performed replacement. If sampling is performed without replacement (as it will invariably be done in practice), the bootstrap will exhibit some bias although it will be positive bias (i.e. the estimate will be larger than the true variance), so that conclusions drawn are conservative. If however, the sampling fraction is small for each stratum the bias is expected to be small ^2, 3^.

**References**

1. Fuller WA. *Sampling statistics*. John Wiley & Sons, 2011.

2. Rao J, Wu C and Yue K. Some recent work on resampling methods for complex surveys. *Survey methodology* 1992; 18: 209-217.

3. Rao JN and Wu C. Resampling inference with complex survey data. *Journal of the American Statistical Association* 1988; 83: 231-241.
